# Efficacy of Manual Therapy and Electrophysical Modalities for Treatment of Cubital Tunnel Syndrome: A Randomized Interventional Trial

**DOI:** 10.3390/life15071059

**Published:** 2025-07-02

**Authors:** Michał Wieczorek, Tomasz Wolny

**Affiliations:** 1 Department of Theoretical and Practical Basics of Physical Therapy, The Jerzy Kukuczka Academy of Physical Education, Mikołowska 72A, 40-065 Katowice, Poland; 2 Musculoskeletal Elastography and Ultrasonography Laboratory, Institute of Physiotherapy and Health Sciences, The Jerzy Kukuczka Academy of Physical Education, Mikołowska 72A, 40-065 Katowice, Poland; t.wolny@awf.katowice.pl

**Keywords:** cubital tunnel syndrome, manual therapy, electrophysical modalities, physiotherapy

## Abstract

The aim of this study was to evaluate the efficacy of manual therapy based on neurodynamic techniques and electrophysical modalities in the conservative treatment of cubital tunnel syndrome (CuTS). A total of 128 upper limbs affected by CuTS were initially enrolled in this study, with 82 completing the full treatment protocol. The participants were divided into the following two intervention arms: the first arm (MT) (42 arms) received therapy based on sliding and tensioning neurodynamic techniques, while the second arm (EM) (40 arms) underwent physiotherapy based on electrophysical modalities, specifically low-level laser therapy (LLLT) and ultrasound therapy (US). Chi^2^ and Student’s *t*-test were used to compare the intervention arms, and no statistically significant differences were found. The evaluated outcomes included nerve conduction testing, ultrasound assessments (measuring cross-sectional area and shear modulus), pain levels, two-point discrimination, thresholds for cutaneous sensory perception, symptom severity, functional ability in specific tasks, and overall post-treatment improvement. Baseline comparisons indicated no statistically significant differences in any measured variables between the intervention groups (*p* > 0.05). Following treatment, each group exhibited significant improvements in their respective parameters (*p* < 0.01). Comparisons between groups post-intervention revealed statistically significant differences in nerve conduction results, ultrasound measurements (cross-sectional area and shear modulus), two-point discrimination, and sensory perception thresholds. These parameters improved more in the MT intervention arm. The use of neurodynamic techniques, ultrasound, and low-level laser therapy in the conservative treatment of mild to moderate forms of CuTS has a beneficial therapeutic effect.

## 1. Introduction

Cubital tunnel syndrome (CuTS) is the second most common peripheral neuropathy of the upper limb, affecting 2–6% of the general population [1,2]. It typically begins with sensory symptom—such as paresthesia and mild hypoesthesia—and eventually progresses to motor deficits, including weakness and atrophy of the hand muscles [1,3]. McGowan classifies CuTS into three stages—mild, moderate, and severe—based on symptom progression [4]. As the condition advances, it can significantly impair daily and professional functioning, making CuTS a relevant medical and socio-economic concern. In a large population-based study of workers in Washington State, the average medical cost of CuTS was estimated at USD 15,200 per case, with an additional USD 19,100 in wage replacement costs, underlining the substantial financial burden associated with the condition [5]. Diagnosing CuTS can be challenging due to anatomical variability and the wide range of symptoms [6]. The causes can include cysts, ganglions, or degenerative changes, and compression can occur at various sites, such as the arcade of Struthers, medial epicondyle, or cubital tunnel, making both diagnosis and treatment selection more complex [7,8]. Although both conservative and surgical treatments are available for CuTS, the optimal approach remains controversial [6,9]. Conservative methods are generally used for mild to moderate cases, with surgery reserved for severe ones, but clear management guidelines are lacking [6,10]. This highlights the need to identify effective and cost-efficient treatment options. We hypothesized that neurodynamic techniques would be more effective than electrophysiological modalities (ultrasound and low-level laser therapy) in improving clinical outcomes in patients with mild to moderate CuTS.

Physiotherapy is a key component of conservative treatment for peripheral neuropathies, but its use in CuTS often relies on clinical experience rather than strong scientific evidence [6]. Systematic and critical reviews have highlighted methodological weaknesses, limited numbers, and inconsistent results across studies [11,12]. To date, only seven randomized trials, three prospective studies, and eight case reports have evaluated physiotherapy for CuTS. The effectiveness of neurodynamic techniques has been examined in three randomized clinical trials [13,14,15] and three case studies [16,17,18]. In most of these studies, neurodynamic techniques were applied as only one component of a broader therapeutic approach, making it difficult to isolate their specific contributions to treatment outcomes. Only one randomized study [13] evaluated neurodynamic techniques as a standalone intervention and demonstrated their potential effectiveness in the treatment of CuTS. Given the limited number of studies in which neurodynamic techniques were used independently, we identified a clear need to further investigate their role. Our study responds to this gap by evaluating neurodynamic techniques as a monotherapy, rather than as part of a multimodal program. This distinction is important, as previous trials often combined neurodynamic techniques with other physiotherapeutic or medical interventions, making it difficult to attribute clinical effects to the techniques themselves. By isolating this variable, our study offers new insight into the specific therapeutic value of neurodynamic mobilization in CuTS and thus strengthens the novelty of our approach within the existing literature. Ultrasound therapy has been assessed in four studies—three of which used it as a standalone treatment and one as part of a combined approach—with reported benefits but no significant differences compared to controls [9,14,15,19]. Similarly, one study comparing low-level laser therapy to ultrasound found no significant difference [19]. These findings highlight the lack of conclusive evidence supporting the effectiveness of these therapies in CuTS treatment. Neurodynamic techniques, ultrasound, and low-level laser therapy have proven effective in treating carpal tunnel syndrome (CTS) [20], prompting investigation into their use for CuTS. Although CTS and CuTS affect different nerves and anatomical regions, both conditions share similar pathophysiological mechanisms—including mechanical compression, impaired axoplasmic transport, and intraneural ischemia—which provides a rationale for evaluating whether interventions effective in treating CTS may also benefit patients with CuTS. A recent randomized, placebo-controlled trial by Wolny et al. [21] further supports the effectiveness of neurodynamic techniques in CuTS treatment, demonstrating significant improvements in clinical and functional outcomes compared to sham therapy. To date, only a limited number of studies—primarily a few randomized controlled trials and case studies—have assessed the effectiveness of physiotherapy for CuTS, highlighting the importance and relevance of our research. This study aimed to compare neurodynamic manual therapy with electrophysical modalities (ultrasound and low-level laser therapy) in treating mild to moderate CuTS. We hypothesized that neurodynamic techniques would be more effective by improving the ulnar nerve’s physiology and biomechanics.

## 2. Materials and Methods

### 2.1. Ethics

Ethical approval for the study was granted by the Bioethics Committee for Scientific Research at the Academy of Physical Education in Katowice on 14 November 2019 (Approval No. 8/2019), in accordance with the principles of the Declaration of Helsinki (1975, revised in 1983). The trial was registered in the Australian New Zealand Clinical Trials Registry (ANZCTR) under the identifier ACTRN12621001623886. The study commenced on 3 January 2022, with final data collection completed by 28 February 2023. All participants were fully informed about the objectives and procedures of the study and were made aware of their right to withdraw at any time without providing a reason. Written informed consent was obtained from each participant prior to enrollment.

### 2.2. Study Design

This study was organized as a single-blinded, randomized, interventional trial at several medical outpatient clinics in Poland’s Silesia region between 2022 and 2023. Blinding was applied to both patients and outcome assessors. Patients were not informed about which treatment was hypothesized to be more effective and were instructed not to discuss their therapy during assessments. All outcome measures were collected by a physiotherapist who was blinded to group allocation and not involved in the treatment process. Eligible participants were consecutive patients diagnosed with CuTS by a physician based on medical history, physical examination, and electrophysiological studies, with the results meeting the inclusion criteria. Only individuals with mild to moderate CuTS—according to the McGowan classification [4]—were included. For bilateral CuTS cases, both hands were assessed and treated. Participants who qualified for the study were randomly assigned to one of two experimental intervention arms—manual therapy (MT) or electrophysical modalities (EM). The MT intervention arm underwent 10 sessions of manual therapy, including sliding and tensioning neurodynamic techniques for the ulnar nerve. The EM intervention arm received 10 treatments combining low-level laser therapy (LLLT) and ultrasound therapy (US). Each therapy was administered five days a week over two weeks, with each session lasting approximately 30 min.

All therapists conducting the intervention were certified in the therapeutic approaches applied. The therapy was delivered in outpatient clinics that adhere to uniform standards of therapeutic practice, ensuring consistency and standardization of treatment across all cases. As a result, the intervention was standardized despite the absence of additional procedures such as session recordings or inter-rater reliability assessments.

Participants did not use any medication specifically for CuTS; they continued only with their regular treatments for pre-existing chronic conditions.

### 2.3. Sample Size

The necessary sample size was estimated based on pilot study results from 10 participants using the sample size calculator [22], with an assumed alpha level of 0.05 and a statistical power of 0.8. The largest required sample size was obtained for measurements employing numerical pain rating scales, specifically nocturnal pain. Based on this analysis, we aimed to recruit about 76 participants (38 for each treatment intervention arm) plus ~20% to account for possible dropouts.

### 2.4. Participants

**Figure 1:**
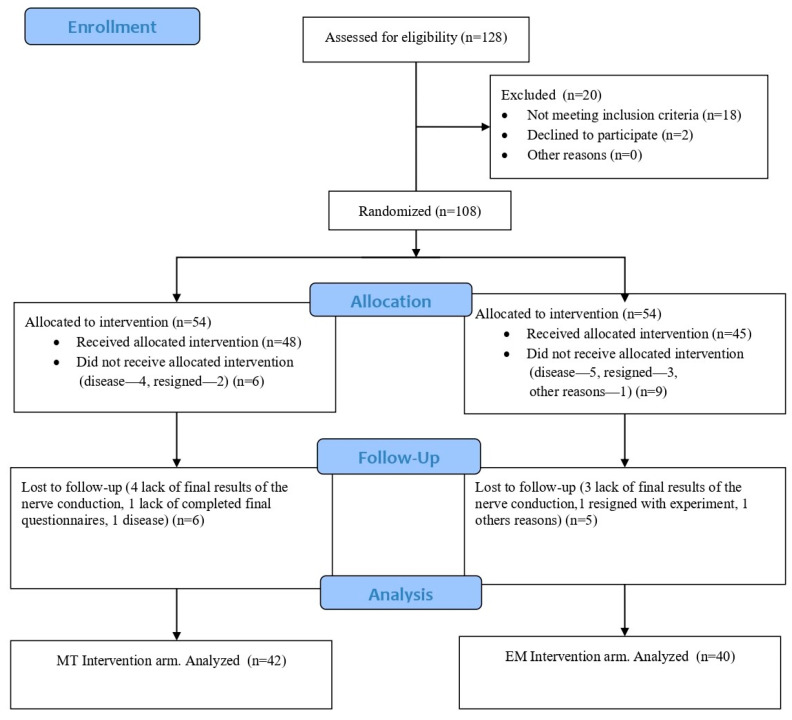
Flow chart. Abbreviations: MT—manual therapy; EM—electrophysical modalities.

Initially, 128 upper limbs affected by CuTS were considered for this study. However, 20 were excluded—18 due to not meeting the inclusion criteria (e.g., McGowan grade III, lack of electrophysiological confirmation, or comorbid neuropathies) and 2 due to participant refusal after initial screening. The remaining limbs were then randomly assigned to either the experimental or control group. During the following stages, 26 limbs were excluded due to illness, participant withdrawal, incomplete final assessments, or other reasons. In total, 82 limbs completed the full study protocol (Figure 1).

Notably, 5 participants in the experimental group and 2 in the control group presented with bilateral CuTS-3.

Cases with missing outcome data were excluded from the analysis, and no imputation methods were applied. Consequently, all statistical analyses were conducted on a per-protocol basis.

It is important to note that in cases of bilateral CuTS, each affected limb was treated as an independent statistical unit. As a result, the total number of limbs analyzed exceeded the number of participants. This approach allowed for more a detailed assessment and preserved the variability between limbs, especially in bilateral cases. The corresponding sample size (*n*) for each analysis is clearly indicated in the relevant tables.

### 2.5. Protocol

#### 2.5.1. Diagnostic Criteria for CuTS

An independent physician established the diagnosis of CuTS based on a comprehensive assessment that included the patient’s medical history, physical examination, and nerve conduction study results. Eligibility for inclusion required the presence of both subjective symptoms, such as pain, numbness, or tingling in the ulnar nerve distribution, and objective clinical signs, including symptom aggravation during elbow flexion, a positive elbow flexion test, a positive Tinel’s sign at the elbow, and classification as grade I or II on the McGowan scale. Additionally, participants had to exhibit sensory abnormalities, detected through two-point discrimination and sensory threshold testing, as well as ultrasound evidence of pathology, such as an increased cross-sectional area or elevated nerve stiffness. All ultrasound measurements were performed by the same experienced sonographer using standardized procedures and identical equipment across all participants, which minimized measurement variability, although inter-rater reliability was not formally assessed. An additional inclusion criterion was the presence of nerve conduction impairment, defined as a motor conduction velocity below 49.3 m/s in the ulnar nerve. This threshold was based on the normative reference values established by the neurophysiology laboratory where all electrophysiological tests were performed. A diagnosis of CuTS was confirmed when at least two of these symptoms were present alongside nerve conduction abnormalities. Additionally, participants had to provide informed consent and have no contraindications to therapy.

Participants were excluded if they showed no symptoms of CuTS; had undergone previous surgical interventions; had a history of mental disorders, cervical radiculopathy, rheumatoid conditions, or diabetes; or were pregnant. Additional exclusion factors included any contraindications to the proposed therapies, lack of cooperation, or unwillingness to take part in the study. To reduce potential confounding, individuals with clinical or electrophysiological signs of cervical radiculopathy or a confirmed diagnosis of diabetes mellitus accompanied by signs or diagnostic evidence of peripheral neuropathy were excluded. Rheumatoid disease was also an exclusion criterion.

#### 2.5.2. Randomization and Allocation

Every successive patient diagnosed with mild to moderate CuTS and meeting the inclusion criteria was admitted into the study. Participants were randomly assigned to one of two parallel experimental intervention arms (MT or EM) using a computer-generated randomization process. A table containing randomly generated numbers ‘1’ and ‘2’ was created, and these numbers were placed in opaque, sealed, and sequentially numbered envelopes, which were then thoroughly shuffled. The envelopes were prepared in advance by an independent researcher who was not involved in administering any of the therapeutic interventions. As each eligible patient progressed to the next stage of the study, they selected an envelope. Those who drew ‘1’ were assigned to the MT intervention arm, while those who drew ‘2’ were allocated to the EM intervention arm. The randomization process was conducted by independent assistants who were not part of the research team and had no involvement in the experiment. No block or stratified randomization was applied. We acknowledge that the absence of stratification by factors such as disease severity may have introduced potential baseline differences between groups. This is addressed as a limitation of the study in the Discussion section.

#### 2.5.3. Blinding Procedures

The clinician who confirmed the diagnosis of CuTS operated independently of the research team and was not involved in the experimental procedures. Diagnostic assessments were completed prior to assigning participants to specific intervention groups. Nerve conduction studies (NCSs) were performed in a separate laboratory under routine clinical protocols, with the lab personnel blinded to the study. The NCS assessments were performed before therapy, after therapy, and again six months later. All additional measurements, as well as supervision and instructions regarding patient-completed questionnaires before therapy, after therapy, and at the six-month follow-up, were handled by physiotherapists who were blinded to the participants’ intervention arm assignments. Because both groups received active therapeutic interventions (manual therapy or electrophysical modalities), participants were not informed which treatment was considered more effective. While the nature of the procedures made full participant blinding infeasible, the intervention conditions were designed to be comparable in structure, duration, and setting. Therefore, the study followed a single-blind design, with outcome assessors blinded to group allocation. The treatment procedures were carried out by physiotherapists who were not part of the research team and had no knowledge of the experimental design or objectives. The treatment followed routine clinical procedures in the facility where the study was conducted. It was carried out by physiotherapists with a minimum of 10 years of experience, including expertise in managing patients with peripheral neuropathies.

### 2.6. Outcome Measures

#### 2.6.1. Primary Outcomes

The ulnar NCS was conducted in an electroneurography laboratory by an experienced neurophysiologist with over 10 years of practice, following a physician’s order. Surface electrodes were used for the study. The examination took place in a room maintained at 24–26 °C. Before the test, patients underwent a 10 to 15min acclimation period. Skin temperature was measured with a surface thermometer and ranged between 32 °C and 34 °C. Assessment of motor conduction velocity was performed at the level of the ulnar groove, in accordance with the reference standards established by the independent neurophysiology laboratory responsible for the NCS. These standards included a motor conduction velocity ≥ 49.3 m/s, a sensory conduction velocity ≥ 54.9 m/s, and a distal motor latency ≤ 3.4 m/s. A reduction in motor conduction velocity was considered the key neurophysiological criterion for diagnosing CuTS. NCS assessments were conducted at the following three time points: baseline (within 1–2 weeks), after treatment (within 1–2 weeks), and at a 6-month follow-up (within 1–2 weeks).

Ultrasound assessments were performed using the Hologic Supersonic Mach 30 system (Supersonic Imagine, Aix En Provence, France) with a linear transducer (5–18 MHz; Super Linear SL18-5, Supersonic Imagine). All procedures—including patient positioning, anatomical landmarks, transducer placement, and measurement techniques—were carried out in accordance with the protocol outlined by Wolny et al. [23]. The cross-sectional area (CSA) and shear modulus of the ulnar nerve were evaluated in four elbow positions—full extension, 45° flexion, 90° flexion, and maximal flexion. It is important to emphasize that the size of the cubital tunnel changes with elbow flexion; therefore, assessing the ulnar nerve at various degrees of elbow flexion is justified, as this may influence both the CSA and the stiffness of the nerve. Measurements were taken within the cubital tunnel, with CSA values recorded in mm^2^ and shear modulus values in kPa. There is currently no established cut-off value for elastography in the assessment of the ulnar nerve. Normative CSA values of the ulnar nerve at the level of the medial epicondyle range from 5.07 to 9.28 mm^2^ [24]. A CSA cut-off value of 10 mm^2^ has been established, above which cubital tunnel syndrome (CuTS) can be diagnosed [25].

All variables were recorded in triplicate, with the mean value of the three trials used for data analysis. Evaluations were performed at three distinct time points—prior to the intervention, immediately following the treatment period, and at a 6-month follow-up.

Pain intensity was measured using the Numerical Pain Rating Scale (NPRS), where 0 denoted no pain and 10 indicated the highest level of pain [26]. Participants were asked to report the most severe pain they had experienced over the past week, both during the day and at night. In cases of bilateral CuTS, each limb was assessed separately. Measurements were taken at the following three time points: prior to the intervention, immediately after treatment, and at the 6-month follow-up

#### 2.6.2. Secondary Outcomes

Static two-point discrimination (2PD) was assessed using the standardized Dellon device (Baseline Discrim-A-Gon Discriminator, Fabrication Enterprises, White Plains, NY, USA), with spike placement guided by the protocol outlined by Wolny et al. [27]. The test was administered on the fourth and fifth digits, applying the discriminator gently to the skin without additional pressure. The minimum distance at which participants reliably distinguished two separate points across three successive attempts was recorded in millimeters for analysis. Assessments were conducted at three time points—prior to treatment, immediately after the intervention, and at the 6-month follow-up. This method has demonstrated high reliability in previous research [27].

Cutaneous sensory perception thresholds (CSPTs) were measured using the TOUCH TEST monofilament kit (North Coast Medical, Inc., Morgan Hill, CA, USA), consisting of five calibrated filaments, each delivering a specific force: green (2.83)—0.07 g; blue (3.61)—0.4 g; purple (4.31)—2.0 g; pink (4.56)—4.0 g; and red (6.65)—300 g. Each filament was applied perpendicularly to the test site, allowing for slight bending, in accordance with the procedure described by Wolny et al. [26]. Assessments were conducted on the fourth and fifth fingers. The thinnest filament consistently detected by the participant in three consecutive applications was recorded for analysis. Scoring was based on the following scale: 0—no sensation; 1—detection of the 0.07 g filament; 2—0.4 g; 3—2.0 g; 4—4.0 g; and 5—300 g [28]. CSPT evaluations were performed at the following three time points: before treatment, immediately after the intervention, and at the 6-month follow-up. Prior research has confirmed the strong reliability of this method [27].

Symptom severity and functional capacity were assessed using the Polish adaptation [29] of the Quick Disabilities of the Arm, Shoulder, and Hand (Q-DASH) questionnaire, a self-report measure consisting of 11 items. The tool evaluates the ability to perform various activities, participation in social roles, limitations in everyday tasks, and symptom intensity both during the day and at night. Responses are rated on a scale from 1 (no difficulty) to 5 (severe difficulty), with higher scores reflecting greater functional impairment. The questionnaire was administered at three time points—prior to the intervention, immediately following treatment, and at the 6-month follow-up.

Neuropathic pain was assessed using the Polish version of the Self-Complete Leeds Assessment of Neuropathic Symptoms and Signs (S-LANSS) questionnaire [30]. The tool comprises seven items, with a maximum total score of 24 points. Higher scores reflect greater neuropathic pain intensity, and a score of 12 or above is indicative of predominantly neuropathic pain. Participants completed the questionnaire at the following three time points: before treatment, immediately after the intervention, and at the 6-month follow-up.

Perceived improvement was measured using the Global Rating of Change (GROC) scale, administered after the intervention and again at the 6-month follow-up. The GROC is a self-report tool that captures the patient’s subjective evaluation of change in their condition. It uses a 15-point scale ranging from −7 (a very great deal worse) to +7 (a very great deal better), with a minimum clinically important difference defined as 3 points [31].

### 2.7. Intervention

During the initial treatment session in both intervention groups (MT and EM), physiotherapists delivered a one-time educational session focused on strategies to minimize symptom aggravation. Patients were advised to avoid positions and activities known to exacerbate CuTS symptoms, such as prolonged elbow flexion, sustained pressure on the elbow, and sleeping with the elbow fully extended.

In the MT group, manual therapy involved the application of neurodynamic techniques targeting the ulnar nerve, specifically utilizing sliding and tensioning approaches. These techniques were executed in two distinct body positions and followed standardized neurodynamic sequences designed for ulnar nerve mobilization, as described below.

- Neurodynamic Approach for the Ulnar Nerve—Variant 1 (NTUN1)

The individual is placed in a supine position. The neurodynamic sequence includes wrist and finger extension, pronation of the forearm, external rotation of the shoulder, elbow flexion, depression of the shoulder girdle, and abduction of the arm. The applied neurodynamic methods involve rhythmic sliding mobilization directed distally through large-range wrist flexion and extension movements. A distal tensioning method is also employed, characterized by small-amplitude wrist motions focused at the end range. Additionally, proximal sliding mobilization is carried out using wide-range flexion and extension of the elbow, while proximal tensioning mobilization uses similar elbow movements but with minimal amplitude near the movement’s endpoint.

b. Neurodynamic Approach for the Ulnar Nerve—Variant 2 (NTUN2)

This technique was also carried out with the participant lying in the supine position. The movement sequence included wrist and finger extension combined with radial deviation, progressing through forearm pronation, internal rotation of the shoulder, full elbow extension, shoulder girdle depression, and arm abduction. Distal sliding mobilization was performed using broad, rhythmic flexion and extension of the wrist and hand, while distal tensioning involved small, controlled movements at the end of the available range. For the elbow segment, proximal sliding was applied through large-range rhythmic flexion and extension, whereas proximal tensioning was delivered using minimal-amplitude movements close to the end range of elbow motion.

The standardized protocol involved one set of 60 repetitions of both distal and proximal sliding and tensioning neurodynamic techniques, performed in each of the two positions (NTUN1 and NTUN2), with 15 s rest intervals between sets. Performing all sliding techniques—both proximal and distal—with the elbow flexed, followed by tension techniques (distal and proximal), also with the elbow flexed, and then repeating the same sequence with the elbow extended and including breaks takes approximately 30 min. The exercises were performed five times per week for a total of 10 sessions. This dosage and structure were based on a recently published randomized, placebo-controlled trial, which used the same number of repetitions in patients with CuTS and demonstrated significant improvements in clinical and neurophysiological outcomes [22].

In the EM intervention arm, physiotherapy was based on electrophysical modalities—low-level laser therapy (LLLT) and ultrasound therapy (US).

- Low-Level Laser Therapy (LLLT)

This treatment was administered with the patient positioned laterally, resting on the side corresponding to the treated limb. The upper limb was positioned with the shoulder girdle in a neutral alignment, the arm adducted and externally rotated, the elbow flexed, and the forearm, wrist, and fingers maintained in a neutral posture. Laser application was performed using the contact technique, targeting the following three specific sites: directly over the cubital tunnel, as well as two centimeters proximally and distally to it. Each session began with exposure to red light delivered by an R650/50 probe operating at 658 nm with an output of 50 milliwatts. This phase of biostimulation lasted for 1 min and 40 s, delivering an energy dose of 5 joules. Subsequently, an infrared laser using the IR810/400 probe was applied, emitting light at 808 nm and 400 milliwatts. This phase lasted 1 min and provided a cumulative dose of 24 joules. The full laser therapy session was completed within a total duration of 8 min.

b. Ultrasound Therapy (US)

In this procedure, the patient was once again placed in a side-lying position on the affected side. The treated limb was positioned with the shoulder girdle held neutrally, the arm adducted and externally rotated, the elbow flexed, and the forearm, wrist, and fingers maintained in a neutral position. Ultrasound was applied using a contact method focused on the cubital tunnel region, extending two centimeters above and below it. The treatment was delivered using a frequency of 1 megahertz, an intensity of 1.0 watts per square centimeter, and a pulsed mode set to 75%. The total application time for each session was 15 min.

Both electrophysical modalities were performed one after the other, with a 5 min break and time for changing the equipment. The therapy was applied five times a week for a total of 10 sessions. The approximate duration of each session was around 30 min.

### 2.8. Statistical Analysis

Statistical analyses were performed using Statistica 13.1 software. Baseline characteristics were compared between intervention groups using independent *t*-tests for continuous variables (age, height, body mass, and BMI) and Chi-squared tests for categorical variables (sex, hand dominance, side of involvement, and whether one or both hands were affected by CuTS). Repeated-measures one-way analysis of variance (ANOVA) was employed to examine changes over time in outcomes—including nerve conduction, cross-sectional area (CSA), shear modulus, pain, two-point discrimination (2PD), cutaneous sensory perception threshold (CSPT), Q-DASH, and S-LANSS scores—across groups. Tukey’s post hoc test was applied to identify between-group differences. Prior to conducting parametric analyses, assumptions of normality and homogeneity of variance were tested using the Shapiro–Wilk and Levene’s tests, respectively, and were found to be satisfied. Statistically significant results are presented as mean differences with corresponding 95% confidence intervals (CIs), and a *p*-value of less than 0.05 was considered indicative of statistical significance.

## 3. Results

### 3.1. Participant Characteristics at Baseline

A total of 82 intervention arms were included in the final analysis—42 in the MT intervention arm and 40 in the EM intervention arm. At the initial assessment, the patients in the two intervention arms were comparable in terms of sex, age, body weight, height, BMI, unilateral or bilateral involvement in CuTS, dominance and laterality of the affected and unaffected hands, and symptom duration. Comprehensive baseline characteristics are summarized in Table 1.

### 3.2. Inter- and Intra-Intervention-Arm Comparisons of Clinical Symptoms

ANOVA performed based on the McGowan classification showed significant differences between intervention arms (*p* < 0.01), a significant treatment effect (*p* < 0.01), and no significant interactions between intervention arms (*p* > 0.05). In the elbow flexion test, ANOVA revealed a group difference, an effect of therapy, and an interaction between the intervention arm and therapy (*p* < 0.01 in all cases). There was no difference between intervention arms and no interaction between the intervention arm and therapy (*p* > 0.05), and the effect of therapy was detected only in the assessment of Tinelʹs sign. The results of Tukey’s post hoc tests and effect sizes are shown in Table 2.

We applied conventional thresholds for interpretation; η^2^ ≤ 0.06 indicates a small effect, >0.06 and ≤0.14 a medium effect, and >0.14 a large effect.

### 3.3. Inter- and Intragroup Comparisons of Primary Outcomes

Repeated-measures ANOVA for ulnar nerve motor conduction velocity revealed a statistically significant main effect of the intervention arm, a significant effect of therapy over time, and a significant interaction between the intervention arm and time point (*p* < 0.01 for all). The observed increase in motor conduction velocity indicates improved neural conduction, which is associated with symptom reduction and enhanced upper limb function.

Repeated-measures ANOVA for CSA in elbow extension and at 45% flexion revealed a significant main effect of the intervention arm, a significant effect of therapy over time, and a significant interaction between the intervention arm and time point (*p* < 0.01 in all cases). For CSA measured at 90% and maximum elbow flexion, no significant differences were found between intervention arms (*p* > 0.05), although both the effect of therapy and the interaction remained statistically significant (*p* < 0.01). Due to the consistency of findings and the volume of data, Table 3 presents the results for 45% and 90% flexion only. The results of Tukey’s post hoc tests and effect sizes are also shown in Table 3. The reduction in CSA may indicate decreased ulnar nerve compression and inflammation, which represent key therapeutic targets in CuTS.

Repeated-measures ANOVA for the ulnar nerve shear modulus at 45% elbow flexion revealed a significant main effect of the intervention arm, a significant effect of therapy over time, and a significant interaction between the intervention arm and time point (*p* < 0.01 in all cases). Comparable results were observed in all other tested elbow positions—extension, 90% flexion, and maximum flexion—with statistically significant outcomes across all effects (*p* < 0.01). Owing to the consistency of findings and the volume of data, Table 3 presents the results for 45% flexion only. Tukey’s post hoc comparisons and effect sizes are also reported in Table 3. The reduction in shear modulus suggests enhanced nerve gliding and decreased stiffness of the surrounding perineural tissues, which may contribute to lowering mechanical irritation during limb movement.

Repeated-measures ANOVA for diurnal and nocturnal pain revealed a significant main effect of the intervention arm (*p* < 0.01), no significant effect of therapy over time (*p* > 0.05), and a significant interaction between the intervention arm and time point (*p* < 0.01). The detailed results of Tukey’s post hoc tests and effect sizes are presented in Table 3. Although the changes in day and night pain varied statistically, they remained closely linked to patients’ functional capacity and overall quality of life.

### 3.4. Inter- and Intragroup Comparisons—Secondary Outcomes

Repeated-measures ANOVA for 2PD and CSPT of the fourth and fifth digits on the affected limb revealed a significant main effect of the intervention arm, a significant effect of therapy over time, and a significant interaction between the intervention arm and time point (*p* < 0.01 in all cases). Due to the consistency and volume of the data, Table 4 presents the results for the fifth finger only. Tukey’s post hoc comparisons and effect sizes are also reported in Table 4. The observed improvements in 2PD and CSPT indicate enhanced sensory discrimination and reduced neural impairment.

Repeated-measures ANOVA for the Q-DASH and S-LANSS questionnaires revealed no significant differences between intervention arms (*p* > 0.05), a significant effect of therapy over time (*p* < 0.01), and no significant interaction between the intervention arm and time point (*p* > 0.05). Tukey’s post hoc comparisons and effect sizes are presented in Table 4. Both questionnaires reflected meaningful subjective improvements—Q-DASH in upper limb function and S-LANSS in neuropathic pain symptoms—with therapeutic effects reaching thresholds of clinical significance.

The results of the GROC scale showed statistically significant improvements after therapy in both intervention arms (*p* < 0.01). The mean improvements in the MT intervention arm were 5.93 (0.98) after therapy and 6.04 (0.92) at the 6-month follow-up. In the EM intervention arm, the mean improvements were 4.66 (0.91) after therapy and 4.91 (0.82) at the 6-month follow-up. In both cases, there were statistically significant differences between intervention arms (*p* < 0.01). Better effects were achieved in the MT intervention arm. The GROC scale showed substantial self-reported improvement, especially in the MT intervention arm, exceeding values typically considered clinically meaningful (improvements > 5 points).

## 4. Discussion

The aim of the present study was to assess the effectiveness of two treatment programs in the conservative management of patients with mild to moderate CuTS. The first therapeutic program used manual therapy based on sliding and tensioning neurodynamic techniques, while the second utilized ultrasound and low-level laser therapy. The results indicated that beneficial effects were obtained after both therapy programs, but the results were slightly better after manual therapy based on neurodynamic techniques. In the intragroup assessment, both intervention arms showed significant improvements in all parameters measured after therapy. In the intergroup comparison, differences were observed in the assessment of nerve conduction, ultrasound parameters (cross-sectional area and shear modulus), two-point discrimination, and cutaneous sensory perception threshold, with better outcomes for the group that underwent therapy based on neurodynamic techniques.

When interpreting the results, we also considered the minimal clinically important difference (MCID), defined according to the approach proposed by Norman et al. [32], based on a threshold of one-half of the standard deviation (½ SD). This allowed for a more clinically meaningful interpretation of the observed changes beyond statistical significance.

In the evaluation of nerve conduction, normative values were not reached in either study group, but an upward trend in this parameter was noted after therapy, suggesting that longer-term therapy might enable normal nerve conduction values to be achieved. The better results obtained in the MT group suggest that improving the biomechanics and physiology of the nerve has a more beneficial effect on nerve regeneration (mean SD = 5.45; MCID~2.8). Svernlöv et al. [13] also observed an improvement in nerve conduction in nine out of twelve patients studied after treatment, and changes also occurred in the group where neurodynamic techniques were used. The difference in outcomes between our study and theirs may partially result from a higher therapeutic dosage in our protocol—60 repetitions per session—compared to a less intensive regimen applied in their study. This increased volume may have contributed to stronger physiological effects on the ulnar nerve. Ozkan et al. [19] found improved nerve conduction in both the ultrasound therapy and low-level laser therapy intervention arms. These findings suggest that a similar therapeutic effect was obtained in our study and that the two therapeutic programs used have a beneficial effect on improving nerve conduction.

In the assessment of ultrasound parameters (cross-sectional area and shear modulus), a statistically significant improvement was found in both intervention arms, with the improvement in shear modulus being greater in the group where neurodynamic techniques were used. This indicates that both therapeutic programs had a beneficial effect on the function of the nerve, but that mechanical intervention using neurodynamic techniques was more effective in increasing nerve elasticity. After therapy, values below 10 mm^2^ were obtained, which is the cut-off value for the ulnar nerve, indicating that normative values were obtained [33]. Nerve flexibility improved after both treatment programs but did not reach normative values [24]. Therefore, longer-term therapy might be necessary for this parameter to reach the values obtained for a healthy ulnar nerve. Unfortunately, to date, no studies have examined the ultrasound parameters of the ulnar nerve after physiotherapy, making it impossible to compare the observed therapeutic effects with the existing literature (cross-section 45°—mean SD = 1.525; MCID~0.76; cross-section 90°—mean SD = 1.57; MCID~0.78; shear modulus 45°—mean SD = 19.15; MCID~9.58). It is important to note that the between-group comparison of the cross-sectional area at 90° elbow flexion did not reveal statistically significant differences (*p* > 0.05). This may suggest that both interventions exert similar effects on the CSA at this specific joint position. The lack of a difference could also be related to mechanical and measurement variability in elbow positioning, particularly at 90°, which may reduce the sensitivity of ultrasound-based comparisons at this angle. We have therefore interpreted the superiority of manual therapy with caution and only in outcomes where statistically significant differences were observed.

There was a significant reduction in both daytime (mean SD = 0.74; MCID~0.37) and nighttime (mean SD = 1.11; MCID~0.55) pain complaints in both study intervention arms. There were no differences between the study intervention arms, yielding a similar treatment effect. Svernlöv et al. [13] reported a similar reduction in daytime and nighttime pain in the neurodynamic therapy group, with no significant differences compared to the group that used orthoses and instruction. Ozkan et al. [19] also achieved a significant reduction in pain in both the ultrasound therapy and low-level laser therapy groups. Galal et al. [15] obtained a significant reduction in pain after a therapeutic program using ultrasound therapy and neurodynamic techniques with additional strengthening exercises. Oskay et al. [16] also obtained a significant reduction in pain after applying sliding and tensioning neurodynamic techniques. Previous studies that have used neurodynamic techniques and ultrasound and low-level laser therapy have shown similar pain reductions to those in our study.

Two-point discrimination sensation (mean SD = 0.64; MCID~0.32) improved in both intervention arms, but a significantly greater effect at long-term follow-up was maintained in the group in which neurodynamic techniques were used. If we adopt the normative values (average 3.34 mm) presented by Louis et al. [34], the values obtained in our study have not yet reached normal values. However, if we consider the values proposed by Silva et al. [35] (ranging from 2 to 5 mm), the average 2PD values obtained in the present study fall within the normal range. Unfortunately, to date, no studies evaluating the effect of neurodynamic techniques, ultrasound therapy, and low-level laser therapy have assessed 2PD sensation, making direct comparisons with other research impossible.

A significant improvement in the cutaneous sensory perception threshold (CSPT) (mean SD = 0.54; MCID~0.27) scores was observed in both intervention arms, with a greater improvement in the neurodynamic therapy group. This improvement persisted in the long-term follow-up. Ozkan et al. [19] obtained a significant improvement in CSPT scores only in the group in which ultrasound therapy was used. There were no significant changes in CSPT scores in the group where therapy was based on low-level laser application. Similarly, Oskay et al. [16] found no change in the CSPT following a treatment program incorporating ultrasound therapy, neurodynamic techniques, and strengthening exercises. Discrepancies between the results obtained may be due to differences in the treatment programs used, but this may be influenced by the small number of individuals in the study by Oskay et al. [16], who conducted a case study with only seven participants.

Upper limb function—as assessed by the Q-DASH scale—improved in both intervention arms, with no intergroup differences (mean SD = 7.59; MCID~3.80). Similar results were obtained in the study by Galal et al. [15] following a therapeutic program using ultrasound therapy and neurodynamic techniques, as well as in a study by Oskay et al. [15], in which cold compresses were included as an additional therapy component.

In the assessment of neuropathic pain using the S-LANSS scale (mean SD = 1.86; MCID~0.93), a similar significant therapeutic effect was obtained in both study intervention arms. However, it is difficult to compare these results with other studies, as only Fernández-de-Las-Peñas et al. [17] have used this scale in their study, which was a single-case study (one patient only). Nevertheless, a beneficial reduction in neuropathic pain was achieved, demonstrating the positive effect of neurodynamic techniques, ultrasound, and low-level laser therapy.

It should also be noted that beneficial effects were observed in the McGowan classification (mean SD = 0.51; MCID~0.26). Furthermore, there was also an increase in asymptomatic time in the elbow flexion test and a decrease in the number of positive Tinel’s signs, which also demonstrates the beneficial effect of the therapeutic programs used.

No studies found in the available literature have evaluated the use of manual therapy based on sliding and tensioning neurodynamic techniques compared to combined ultrasound and low-level laser therapy. Therefore, the present study is the first of its kind. The positive therapeutic results obtained show that the conservative treatment of mild to moderate CuTS using physiotherapy can be effective, both immediately and in the long term. There is a need for many more similar randomized clinical trials, preferably at different research centers, to consolidate this knowledge. At this stage, it is difficult to compare the therapeutic effects achieved with those in other studies, as all studies conducted to date have used varying treatment programs. Svernlöv et al. [13] compared a group using an orthosis, a group receiving neurodynamic techniques, and a group provided with only adequate instruction. After therapy, there were no significant differences between the groups. Ozkan et al. [19] evaluated the effectiveness of ultrasound therapy compared to low-level laser therapy. Significant treatment effects were obtained, yet there were no intergroup differences. In their study, Gaber et al. [14] used neurodynamic techniques and ultrasound therapy, reporting significant improvements in grip strength. Galal et al. [15] used ultrasound therapy, neurodynamic techniques, and strengthening exercises, achieving improvements in pain reduction, muscle strength, and upper limb function. The advantage of these studies is that they are randomized clinical trials. Unfortunately, methodological differences make direct comparisons difficult. Other studies that used neurodynamic techniques or ultrasound and low-level laser therapy have been case studies with small sample sizes, so it is difficult to draw far-reaching conclusions based on them [16,18].

The implemented therapy protocol, which incorporated sliding and tensioning neurodynamic techniques, yielded positive outcomes in both objective measurements and subjective assessments by patients with CuTS, supporting its suitability for routine clinical application. The observed therapeutic effects may be attributed to reductions in intra- and extra-neural edema, enhanced vascular perfusion, and the restoration of nerve tissue mobility and elasticity. Neurodynamic techniques have also been reported to facilitate axoplasmic flow, increase motor unit activation, enhance muscle strength, and reduce pain intensity [36,37]. Maintaining adequate axonal transport and neural mobility is vital for preserving the structural and functional integrity of neurons—outcomes that can be promoted through both sliding and tensioning approaches. Notably, a rebound in shear modulus values was observed in the EM intervention arm during follow-up. This increase may suggest the limited persistence of EM-related effects, possibly resulting from insufficient neuromechanical adaptation or the absence of continued home-based exercises. It may also reflect the transient nature of EM-induced responses, such as short-term analgesia or soft tissue changes, which tend to diminish over time.

In contrast, the beneficial therapeutic effects of ultrasound and low-level laser therapy may be explained by the fact that ultrasound promotes remyelination and axonal proliferation, in addition to suppressing the expression of pro-inflammatory genes and nerve growth inhibitors [38]. At the same time, the thermal and non-thermal effects of ultrasound on tissues can be observed [39]. Low-level laser therapy, on the other hand, facilitates the healing of soft tissue, nerve tissue, and bone. It has also been shown to influence nerve conduction. The mechanisms underlying these effects are macrophage stimulation, mast cell degranulation, fibroblast activation, angiogenesis, increased phagocytic activity, and the photodissociation of oxyhemoglobin. Nerve tissue has demonstrated increases in both conduction velocity and action potential in response to laser stimulation [40].

Although surgical decompression remains the standard treatment for moderate to severe CuTS, its outcomes can vary, and postoperative complications such as nerve instability, scarring, or incomplete symptom resolution are not uncommon. A recent meta-analysis reported improvements in approximately 87% of patients undergoing in situ decompression, with a 95% CI of 82–91% [41].

In a classic prospective series using minimal incision in situ decompression, the outcomes were classified as “excellent” or “good” in up to 93% of cases [41]. In a randomized study comparing simple decompression with anterior transposition, the authors found no significant difference in outcomes, with success rates ranging from 70 to 90% [42,43,44,45,46,47,48]. These results align with earlier observations that persistent symptoms occurred in about 7% of patients after decompression alone.

In light of these findings, our results suggest that conservative management—particularly neurodynamic manual therapy—can provide meaningful clinical benefits in mild to moderate CuTS without the risks associated with surgery. Although manual therapy demonstrated significantly greater improvements in several outcomes, both intervention arms led to relevant clinical improvements, and some parameters showed no difference between therapies. These results support the use of both conservative approaches in clinical practice, depending on the patient profile and therapeutic goals.

Although the between-group difference in GROC scores reached statistical significance, the clinical relevance of this difference should be interpreted with caution due to the subjective nature of the scale.

While manual therapy showed greater improvements in structural and functional nerve parameters, it is important to note that pain reduction—a key patient-centered outcome—was similar in both groups. This suggests that both interventions offer comparable benefits in relieving subjective discomfort, despite differences in physiological response.

Furthermore, although some participants withdrew from the study, the dropout rate was comparable between intervention arms, and only those who completed the full protocol were included in the final analysis. This approach minimizes the potential impact of attrition bias on the study’s internal validity.

A key strength of this study lies in its originality, as it is the first to directly compare the effects of sliding and tensioning neurodynamic techniques with a therapeutic approach involving ultrasound and low-level laser therapy. Another notable advantage is the clearly defined intervention protocol, which can be readily applied in everyday clinical settings. The inclusion of a relatively large sample of individuals with CuTS further enhances the value of the findings. Existing research supports the effectiveness of both neurodynamic techniques and interventions incorporating ultrasound and low-level laser therapy in the management of mild to moderate CuTS. Future investigations should aim to assess the potential benefits of combining these therapeutic approaches into a single, integrated treatment protocol.

## 5. Study Limitations

The main limitation of this study is the lack of control intervention arms with sham therapy. Additionally, it remains to be tested whether a combined therapeutic approach—neurodynamic techniques together with ultrasound and low-level laser therapy—would be more beneficial in improving the condition of people with CuTS. A double-blind study would also be valuable to strengthen these findings. Moreover, the absence of a sham control arm limits the ability to isolate specific treatment effects from non-specific influences, such as therapist interactions and patient expectations. These placebo-related factors may have partially contributed to the observed improvements. It should also be acknowledged that the instruction to avoid activities that provoke symptoms constitutes a therapeutic intervention in itself and may have contributed to the observed improvements in both intervention arms.

## 6. Clinical Implications

Manual therapy should be considered a first-line conservative treatment for mild to moderate CuTS.

It showed superior outcomes in nerve conduction, ultrasound parameters (cross-sectional area and shear modulus), two-point discrimination, and sensory perception thresholds.

Electrophysical modalities (ultrasound and low-level laser therapy) remain a valid and effective alternative, especially for patients who are not suitable candidates for manual therapy.

Neurodynamic techniques appear to produce measurable structural and functional changes in the ulnar nerve, including the following:

- A reduction in nerve swelling or compression, as suggested by decreased cross-sectional area (CSA); however, this remains a hypothetical mechanism and requires validation through advanced imaging such as MRI;
- Increased nerve elasticity and mobility;
- Enhanced axoplasmic flow and nerve regeneration.

Neurodynamic therapy may be more effective in improving fine sensory discrimination and tactile perception, which are critical for hand dexterity and functional tasks in daily life.

Patient education on avoiding symptom-provoking activities (e.g., prolonged elbow flexion, leaning on the elbow, improper sleep positioning) should be considered an essential part of CuTS management, as it likely contributes significantly to symptom improvement.

Clinicians should consider implementing standardized neurodynamic protocols in daily physiotherapy practice for patients with CuTS, given their demonstrated safety, efficacy, and ease of application.

## 7. Conclusions

The use of neurodynamic techniques, ultrasound, and low-level laser therapy in the conservative treatment of mild to moderate forms of CuTS appears to have a beneficial therapeutic effect. Slightly better results were obtained in the intervention arm in which neurodynamic techniques were used, particularly in improving nerve conduction, ultrasound parameters (cross-sectional area and shear modulus), two-point discrimination, and the cutaneous sensory perception threshold. However, it is important to note that differences in some key outcome measures—such as the Q-DASH and S-LANSS scores—were not statistically significant. Pain intensity and upper limb function improved similarly in both intervention arms, suggesting that electrophysical modalities (ultrasound and low-level laser therapy) were comparably effective in relieving discomfort and improving functional capacity. Therefore, both interventions appear to offer meaningful benefits, and the choice of treatment may be tailored to the individual patient’s characteristics and clinical context. While certain findings suggest potential advantages of neurodynamic techniques, the results do not support a definitive conclusion regarding their overall superiority.

## Figures and Tables

**Table 1:** Between-intervention-arm comparisons of participants’ characteristics at baseline.

Characteristics	MT Intervention Arm (n = 42)	EM Intervention Arm (n = 40)	*p*-Value
Women/Men (n, %)	13 (31%)/29 (69%)	15 (37%)/25 (63%)	0.5319 ^a^
Age (mean, SD, min-max), year	54.2; 7.48; 40–68	52; 7.04; 41–68	0.1677 ^b^
Body mass (mean, SD, min-max), kg	77.1; 13.1; 49–102	73.5; 12.1; 49–102	0.1967 ^b^
Body height (mean, SD, min-max), cm	174; 10.7; 148–189	172; 9.98; 148–188	0.4747 ^b^
BMI (mean, SD, min-max), kg/m^2^	25.3; 3.51; 20.2–35.8	24.8; 2.68; 20.8–35.8	0.4502 ^b^
CuTS unilateral/bilateral (n, %)	39 (93%)/3 (7%)	38 (95%)/2 (5%)	0.8233 ^a^
Symptomatic hand right/left (n, %)	21 (47%)/24 (53%)	18 (43%)/24 (57%)	0.7211 ^a^
Asymptomatic hand right/left (n, %)	21 (52%)/19 (48%)	22 (58%)/16 (42%)	0.6321 ^a^
Dominant hand right/left (n, %)	37 (88%)/5 (12%)	34 (85%)/6 (15%)	0.6811 ^a^
Symptoms duration (mean, SD, min-max), month	10.9; 2.99; 6–18	11.4; 2.68; 7–18	0.4176 ^b^

Abbreviations: MT—manual therapy; EM—electrophysical modalities; BMI—body mass index; n—number of people; %—percentage value; SD—standard deviation; ^a^ Chi—square test; ^b^ Student’s *t*-test.

**Table 2:** Inter- and intra-intervention-arm comparisons—clinical symptoms.

Clinical Tests	Group	MT Intervention Arm (n = 42)		EM Intervention Arm (n = 40)		Inter-Intervention-Arm Difference p [η^2^]; 95% CI
	Time Point	Mean (SD)	Intragroup Differences p [η^2^]	Mean (SD)	Intragroup Differences p [η^2^]	
McGowan classification (scale 1–3)	Baseline Final Six-month follow-up	1.44 (0.51) 0.44 (0.51) 0.33 (0.52)	B vs. F—0.0000 * [0.79] B vs. S—0.0000 [0.75] F vs. S—0.7941 [0.03]	1.54 (0.51) 0.76 (0.69) 0.66 (0.65)	B vs. F—0.0000 * [0.66] B vs. S—0.0000 * [0.69] F vs. S—0.8961 [0.03]	0.9608 [0.01] −0.31 to 0.11 0.1041 [0.07] −0.57 to −0.06 0.0751 [0.08] −0.58 to −0.08
Elbow flexion test (0–60 s)	Baseline Final Six-month follow-up	22.1 (6.59) 40.7 (13.1) 46.1 (15.4)	B vs. F—0.0000 * [0.75] B vs. S—0.0000 * [0.75] F vs. S—0.0235 * [0.22]	20.1 (4.46) 29.3 (6.82) 43.8 (13.1)	B vs. F—0.0000 * [0.67] B vs. S—0.0000 * [0.81] F vs. S—0.0000 * [0.75]	0.1796 [0.04] −0.42 to 4.41 0.0000 * [0.23] 6.91 to 15.97 0.9281 [0.01] −8.57 to 3.71
Tinelʹs sign (0—negative; 1—positive)	Baseline Final Six-month follow-up	0.71 (0.45) 0.2 (0.4) 0.13 (0.34)	B vs. F—0.0000 * [0.51] B vs. S—0.0000 * [0.54] F vs. S—0.9621 [0.02]	0.64 (0.48) 0.35 (0.48) 0.28 (0.45)	B vs. F—0.0077 * [0.21] B vs. S—0.0002 * [0.23] F vs. S—0.9561 [0.02]	0.9808 [0.01] −0.13 to 0.26 0.5755 [0.03] −0.34 to −0.03 0.6085 [0.03] −0.32 to 0.01

Abbreviations: MT—manual therapy; EM—electrophysical modalities; n—number of people; p—significance level; SD—standard deviation; CI—confidence interval; B—baseline; F—final; S—six-month follow-up; * statistically significant difference; η^2^—effect size (eta squared).

**Table 3:** Inter- and intra-intervention-arm comparisons—primary outcomes.

Clinical Tests	Group	MT Intervention Arm (n = 42)		EM Intervention Arm (n = 40)		Inter-Intervention-Arm Difference p [η^2^]; 95% CI
	Time Point	Mean (SD)	Intragroup Differences p [η^2^]	Mean (SD)	Intragroup Differences p [η^2^]	
Motor conduction velocity (m/s)	Baseline Final Six-month follow-up	35.1 (5.78) 43.6 (7.29) 45.2 (7.23)	B vs. F—0.0000 * [0.79] B vs. S—0.0000 * [0.73] F vs. S—0.2223 [0.07]	36.5 (5.12) 38.8 (4.62) 40.1 (4.41)	B vs. F—0.0206 * [0.42] B vs. S—0.0000 * [0.43] F vs. S—0.4421 [0.13]	0.8707 [0.02] −0.37 to 0.88 0.0021 * [0.14] 2.24 to 7.51 0.0011 * [0.15] 2.52 to 7.67
Ultrasound cross-sectional area 45° flexion (mm^2^)	Baseline Final Six-month follow-up	10.9 (1.54) 9.14 (1.41) 8.88 (1.31)	B vs. F—0.0000 * [0.74] B vs. S—0.0000 * [0.80] F vs. S—0.2544 [0.11]	11.1 (1.51) 10.7 (1.26) 10.3 (1.14)	B vs. F—0.0095 * [0.41] B vs. S—0.0000 * [0.55] F vs. S—0.0096 * [0.36]	0.9725 [0.01] −0.88 to 0.42 0.0001 * [0.26] −1.01 to 15.97 0.0001 * [0.26] −1.95 to −0.91
Ultrasound cross-sectional area 90° flexion (mm^2^)	Baseline Final Six-month follow-up	10.4 (1.58) 10.1 (1.51) 9.74 (1.47)	B vs. F—0.0000 * [0.58] B vs. S—0.0000 * [0.78] F vs. S—0.0000 * [0.75]	10.6 (1.55) 9.91 (1.45) 9.59 (1.32)	B vs. F—0.0000 * [0.84] B vs. S—0.0000 * [0.86] F vs. S—0.0000 * [0.49]	0.9158 [0.00] −0.81 to 0.52 0.9955 [0.00] −0.47 to 0.79 0.9998 [0.00] −0.44 to 0.75
Ultrasound shear modulus 45° flexion (kPa)	Baseline Final Six-month follow-up	91.6 (20.1) 53.7 (15.6) 42.1 (12.1)	B vs. F—0.0000 * [0.89] B vs. S—0.0000 * [0.91] F vs. S—0.0000 * [0.55]	88.2 (17.2) 74.3 (15.4) 72.1 (12.6)	B vs. F—0.0000 * [0.72] B vs. S—0.0000 * [0.73] F vs. S—0.7842 [0.11]	0.9254 [0.01] −4.63 to 11.3 0.0000 * [0.31] −27.2 to −14.1 0.0000 * [0.60] −35.3 to −24.7
Diurnal pain NPRS (0—no pain; 10—maximum pain)	Baseline Final Six-month follow-up	2.57 (0.69) 0.82 (0.74) 0.37 (0.49)	B vs. F—0.0000 * [0.86] B vs. S—0.0000 * [0.87] F vs. S—0.0021 * [0.25]	2.61 (0.79) 0.83 (0.76) 0.59 (0.73)	B vs. F—0.0000 * [0.87] B vs. S—0.0000 * [0.85] F vs. S—0.3665 [0.08]	0.9998 [0.00] −0.35 to 0.27 0.9898 [0.00] −0.33 to 0.31 0.7231 [0.03] −0.48 to 0.04
Nocturnal pain NPRS (0—no pain; 10—maximum pain)	Baseline Final Six-month follow-up	4.73 (1.12) 1.11 (0.91) 0.57 (0.54)	B vs. F—0.0000 * [0.96] B vs. S—0.0000 * [0.95] F vs. S—0.0013 * [0.29]	4.69 (1.09) 1.35 (1.18) 0.54 (0.71)	B vs. F—0.0000 * [0.93] B vs. S—0.0000 * [0.94] F vs. S—0.0000 * [0.40]	0.9999 [0.00] −0.41 to 0.49 0.8321 [0.01] −0.69 to 0.21 0.9998 [0.00] −0.23 to 0.29

Abbreviations: MT—manual therapy; EM—electrophysical modalities; NPRS—Numerical Pain Rating Scale; m/s—meter per second; mm^2^—square millimeters; kPa—kilopascals; n—number of people; p—significance level; SD—standard deviation; CI—confidence interval; B—baseline; F—final; S—six-month follow-up; * statistically significant difference and intragroup comparison of secondary outcomes; η^2^—effect size (eta squared).

**Table 4:** Inter- and intra-intervention-arm comparisons—secondary outcomes.

Clinical Tests	Group	MT Intervention Arm (n = 42)		EM Intervention Arm (n = 40)		Inter-Intervention-Arm Difference p [η^2^]; 95% CI
	Time Point	Mean (SD)	Intragroup Differences p [η^2^]	Mean (SD)	Intragroup Differences p [η^2^]	
2PD—5th finger (mm)	Baseline Final Six-month follow-up	7.07 (0.66) 5.81 (0.71) 4.66 (0.87)	B vs. F—0.0000 * [0.74] B vs. S—0.0000 * [0.82] F vs. S—0.0000 * [0.52]	7.17 (0.62) 6.16 (1.05) 5.64 (1.03)	B vs. F—0.0000 * [0.59] B vs. S—0.0000 * [0.74] F vs. S—0.0035 * [0.40]	0.9916 [0.01] −0.38 to 0.16 0.3689 [0.09] −0.74 to 0.02 0.0000 * [0.21] −1.39 to −0.57
CSPT—5th finger (scale 0–4)	Baseline Final Six-month follow-up	3.11 (0.52) 1.82 (0.61) 1.47 (0.55)	B vs. F—0.0000 * [0.75] B vs. S—0.0000 * [0.83] F vs. S—0.0007 * [0.24]	3.12 (0.55) 2.56 (0.55) 2.57 (0.55)	B vs. F—0.0000 * [0.56] B vs. S—0.0000 * [0.59] F vs. S—0.9996 [0.00]	0.9999 [0.00] −0.23 to 0.23 0.0000 * [0.29] −0.98 to −0.48 0.0000 * [0.50] −1.34 to −0.86
Q-DASH (scale 0–55)	Baseline Final Six-month follow-up	52.2 (8.22) 4.08 (3.07) 1.91 (2.85)	B vs. F—0.0000 * [0.97] B vs. S—0.0000 * [0.98] F vs. S—0.1065 [0.42]	52.4 (6.96) 4.31 (3.31) 2.41 (2.79)	B vs. F—0.0000 * [0.99] B vs. S—0.0000 * [0.99] F vs. S—0.2589 [0.36]	0.9999 [0.00] −3.48 to 3.04 0.9998 [0.00] −1.58 to 1.14 0.9975 [0.00] −1.71 to 0.71
S-LANSS (scale 0–24)	Baseline Final Six-month follow-up	13.7 (1.84) 2.93 (2.78) 2 (2.52)	B vs. F—0.0000 * [0.98] B vs. S—0.0000 * [0.97] F vs. S—0.0045 * [0.18]	13.8 (1.88) 3.04 (2.62) 1.91 (2.16)	B vs. F—0.0000 * [0.98] B vs. S—0.0000 * [0.98] F vs. S—0.0003 * [0.36]	0.9994 [0.00] −0.96 to 0.62 0.9999 [0.00] −1.26 to 1.04 0.9999 [0.00] −0.91 to 1.11

Abbreviations: MT—manual therapy; EM—electrophysical modalities; 2PD—two-point discrimination sense; CSPT—cutaneous sensory perceptron threshold; Q-DASH—Quick Disabilities of Arm Shoulder and Hand; S-LANSS—Leeds Assessment of Neuropathic Symptoms and Signs; n—number of people; p—significance level; SD—standard deviation; CI—confidence interval; B—baseline; F—final; S—six-month follow-up; * statistically significant difference; η^2^—effect size (eta squared).

## Data Availability

The data presented in this study are available upon reasonable request from the corresponding author due to privacy reasons.

## Abbreviations

The following abbreviations are used in this manuscript:

S-LANSS	Self-Complete Leeds Assessment of Neuropathic Symptoms and Signs
NTUN1	Neurodynamic Techniques for the Ulnar Nerve 1
NTUN2	Neurodynamic Techniques for the Ulnar Nerve 2
Q-DASH	Quick Disabilities of the Arm, Shoulder, and Hand
ANOVA	One-Way Analysis of Variance
CSPT	Cutaneous Sensory Perception Threshold
CuTS	Cubital Tunnel Syndrome
LLLT	Low-Level Laser Therapy
GROC	Global Rating of Change
MCID	Minimal Clinically Important Difference
CSA	Cross-Sectional Area
NCS	Nerve Conduction Studies
BMI	Body Mass Index
2PD	Static Two-Point Discrimination
US	Ultrasound Therapy
MT	Manual Therapy
EM	Electrophysical Modalities
CI	Confidence Interval

## References

[1] Elhassan B., Steinmann S.P. (2007). Entrapment neuropathy of the ulnar nerve. J. Am. Acad. Orthop. Surg..

[2] An T.W., Evanoff B.A., Boyer M.I., Osei D.A. (2017). The Prevalence of Cubital Tunnel Syndrome: A Cross-Sectional Study in a U.S. Metropolitan Cohort. J. Bone Jt. Surg..

[3] Kooner S., Cinats D., Kwong C., Matthewson G., Dhaliwal G. (2019). Conservative treatment of cubital tunnel syndrome: A systematic review. Orthop. Rev..

[4] McGowan J. (1950). The results of transposition of the ulnar nerve for traumatic ulnar neuritis. J. Bone Jt. Surg. Br..

[5] Juratli S.M., Nayan M., Fulton-Kehoe D., Robinson L.R., Franklin G.M. (2010). A Population-Based Study of Ulnar Neuropathy at the Elbow in Washington State Workers’ Compensation. Am. J. Ind. Med..

[6] Mezian K., Jacisko J., Kaiser R., Machac S., Steyerová P., Sobotová K., Angerová Y., Nanka O. (2021). Ulnar Neuropathy at the Elbow: From Ultrasound Scanning to Treatment. Front. Neurol..

[7] Assmus H., Antoniadi G., Bischoff C., Hoffmann R., Martini A.K., Preiler P., Scheglmann K., Schwerdtfeger K., Wessels K.D., Wüstner-Hofmann M. (2011). Cubital Tunnel Syndrome a Review and Management Guidelines. Zentralbl. Neurochir..

[8] Ochiai N., Honmo J., Tsujino A., Nisiura Y. (2000). Electrodiagnosis in Entrapment Neuropathy by the Arcade of Struthers. Clin. Orthop. Relat. Res..

[9] Cutts S. (2007). Cubital Tunnel Syndrome. Postgrad. Med. J..

[10] Palmer B.A., Hughes T.B. (2010). Cubital tunnel syndrome. J. Hand Surg. Am..

[11] Wolny T., Fernández-de-las Peñas C., Buczek T., Domin M., Granek A., Linek P. (2022). The Effects of Physiotherapy in the Treatment of Cubital Tunnel Syndrome: A Systematic Review. J. Clin. Med..

[12] Wieczorek M., Gnat R., Wolny T. (2024). The Use of Physiotherapy in the Conservative Treatment of Cubital Tunnel Syndrome: A Critical Review of the Literature. Diagnostics.

[13] Svernlöv B., Larsson M., Rehn K., Adolfsson L. (2009). Conservative treatment of the cubital tunnel syndrome. J. Hand Surg..

[14] Gaber M.R. (2021). Ultrasound versus Nerve Gliding on Hand Grip Strength in Cubital Tunnel Syndrome. Egypt. J. Phys. Ther..

[15] Galal D.O.S.M.G., Abdelmageed S.M., Elserty N., Helmy A.M. (2021). Effect of Dry Cupping Therapy with Neurodynamic Mobilization on Pain Intensity, Muscle Strength and Functional Abilities in Patients with Cubital Tunnel Syndrome: A Randomized Clinical Trial. Turk. J. Physiother. Rehabil..

[16] Oskay D., Meriç A., Nuray K., Firat T., Ayhan C., Leblebicioglu G. (2010). Neurodynamic mobilization in the conservative treatment of cubital tunnel syndrome: Long-term follow-up of 7 cases. J. Manip. Physiol. Ther..

[17] Fernández-de-Las-Peñas C., Arias-Buría J.L., El Bachiri Y.R., Plaza-Manzano G., Cleland J.A. (2020). Ultrasound-guided percutaneous electrical stimulation for a patient with cubital tunnel syndrome: A case report with a one-year follow-up. Physiother. Theory Pract..

[18] Coppieters M.W., Bartholomeeusen K.E., Stappaerts K.H. (2004). Incorporating nerve-gliding techniques in the conservative treatment of cubital tunnel syndrome. J. Manip. Physiol. Ther..

[19] Ozkan F.U., Saygı E.K., Senol S., Kapcı S., Aydeniz B., Aktas I., Gozke E. (2015). New Treatment Alternatives in the Ulnar Neuropathy at the Elbow: Ultrasound and Low-Level Laser Therapy. Acta Neurol. Belg..

[20] Wolny T., Linek P. (2018). Neurodynamic Techniques Versus “Sham” Therapy in the Treatment of Carpal Tunnel Syndrome: A Randomized Placebo-Controlled Trial. Arch. Phys. Med. Rehabil..

[21] Wolny T., Wieczorek M. (2025). Real Versus Sham-Based Neurodynamic Techniques in the Treatment of Cubital Tunnel Syndrome: A Randomized Placebo-Controlled Trial. J. Clin. Med..

[22] Arifin W.N. Sample Size Calculator. http://wnarifin.github.io.

[23] Wolny T., Fernández-De-Las-Peñas C., Granek A., Linek P. (2022). Changes in Ultrasound Measurements of the Ulnar Nerve at Different Elbow Joint Positions in Patients with Cubital Tunnel Syndrome. Sensors.

[24] Jacob D., Creteur V., Courthaliac C., Bargoin R., Sassus B., Bacq C., Rozies J.L., Cercueil J.P. (2004). Sonoanatomy of the Ulnar Nerve in the Cubital Tunnel: A Multicentre Study by the GEL. Eur. Radiol.

[25] Wiesler E.R., Chloros G.D., Cartwright M.S., Shin H.W., Walker F.O. (2006). Ultrasound in the Diagnosis of Ulnar Neuropathy at the Cubital Tunnel. J. Hand Surg. Am..

[26] Jensen M.P., Turner J.A., Romano J.M., Fisher L.D. (1999). Comparative reliability and validity of chronic pain intensity measures. Pain.

[27] Wolny T., Fernández-De-Las-Peñas C., Granek A., Linek P. (2022). Reliability of Ulnar Nerve Sensation Tests in Patients with Cubital Tunnel Syndrome and Healthy Subjects. Diagnostics.

[28] Schreuders T.A., Selles R.W., van Ginneken B.T., Janssen W.G., Stam H.J. (2008). Sensory evaluation of the hands in patients with Charcot-Marie-Tooth disease using Semmes-Weinstein monofilaments. J. Hand Ther..

[29] Golicki D., Krzysiak M., Strzelczyk P. (2013). Translation and Cultural Adaptation of the Polish Version of “Disabilities of the Arm, Shoulder and Hand” (DASH) and Quickdash Questionnaires. Value Heal..

[30] Cnotliwy M., Jurewicz A., Janowska-Gołąb M., Kazimierczak A., Głazek W. (2016). The Self-Complete of Leeds Assessment Neuropathic Symptoms and Signs: An attempt to adapt it for Polish population. Pomeranian J. Life Sci..

[31] Jaeschke R., Singer J., Guyatt G.H. (1989). Measurement of health status: Ascertaining the minimal clinically important difference. Control. Clin. Trials..

[32] Norman G.R., Sloan J.A., Wyrwich K.W. (2003). Interpretation of Changes in Health-Related Quality of Life. Med. Care.

[33] Chang K.V., Wu W.T., Han D.S., Özçakar L. (2018). Ulnar Nerve Cross-Sectional Area for the Diagnosis of Cubital Tunnel Syndrome: A Meta-Analysis of Ultrasonographic Measurements. Arch. Phys. Med. Rehabil..

[34] Louis D.S., Greene T.L., Jacobson K.E., Rasmussen C., Kolowich P., Goldstein S.A. (1984). Evaluation of normal values for stationary and moving two-point discrimination in the hand. J. Hand Surg. Am..

[35] Silva P.G., Jones A., Araujo P.M.P., Natour J. (2014). Assessment of light touch sensation in the hands of systemic sclerosis patients. Clinics.

[36] Nee R.J., Butler D. (2006). Management of Peripheral Neuropathic Pain: Integrating Neurobiology, Neurodynamics, and Clinical Evidence. Phys. Ther. Sport..

[37] Villafañe J.H., Silva G.B., Fernandez-Carnero J. (2011). Short-Term Effects of Neurodynamic Mobilization in 15 Patients with Secondary Thumb Carpometacarpal Osteoarthritis. J. Manip. Physiol. Ther..

[38] Ito A., Wang T., Nakahara R., Kawai H., Nishitani K., Aoyama T., Kuroki H. (2020). Ultrasound therapy with optimal intensity facilitates peripheral nerve regeneration in rats through suppression of pro-inflammatory and nerve growth inhibitor gene expression. PLoS ONE.

[39] Baker K.G., Robertson V.J., Duck F.A.A. (2001). Review of Therapeutic Ultrasound: Biophysical Effects. Phys. Ther..

[40] Kitchen S.S., Partridge C.J. (1991). A Review of Low Level Laser Therapy: Part I: Background, Physiological Effects and Hazards. Physiotherapy.

[41] Wade R.G., Griffiths T.T., Flather R., Burr N.E., Teo M., Bourke G. (2020). Safety and Outcomes of Different Surgical Techniques for Cubital Tunnel Decompression. JAMA Netw. Open.

[42] Starownik J., Szewczyk A., Kołpaczyńska S., Niedobylski S., Bartoszek L., Orłowska D., Wójcik A. (2024). Primary Cubital Tunnel Syndrome–Surgical Treatment Methods and Their Effectiveness. Qual. Sport.

[43] Kessler R.B., Thompson R.G., Lourie G.M. (2020). Cubital Tunnel Syndrome: A Surgical Modification to in Situ Decompression to Improve Results. JSES Int..

[44] Yahya A., Malarkey A.R., Eschbaugh R.L., Bamberger H.B. (2018). Trends in the Surgical Treatment for Cubital Tunnel Syndrome: A Survey of Members of the American Society for Surgery of the Hand. Hand.

[45] Xie Q., Shao X., Song X., Wang F., Zhang X., Wang L., Zhang Z., Lyu L. (2022). Ulnar Nerve Decompression and Transposition with versus without Supercharged End-to-Side Motor Nerve Transfer for Advanced Cubital Tunnel Syndrome: A Randomized Comparison Study. J. Neurosurg..

[46] Fok M.W.M., Cobb T., Bain G.I. (2021). Endoscopic Cubital Tunnel Decompression – Review of the Literature. J. Orthop. Surg..

[47] Carlton A., Khalid S.I. (2018). Surgical Approaches and Their Outcomes in the Treatment of Cubital Tunnel Syndrome. Front. Surg..

[48] Goldfarb C.A., Sutter M.M., Martens E.J., Manske P.R. (2009). Incidence of Re-Operation and Subjective Outcome Following in Situ Decompression of the Ulnar Nerve at the Cubital Tunnel. J. Hand Surg..

